# The Online Health Information–Seeking Behaviors of People Who Have Experienced Stroke: Qualitative Interview Study

**DOI:** 10.2196/54827

**Published:** 2024-10-18

**Authors:** Brigid Clancy, Billie Bonevski, Coralie English, Ashleigh Guillaumier

**Affiliations:** 1 School of Medicine and Public Health College of Health, Medicine and Wellbeing The University of Newcastle Callaghan Australia; 2 Hunter Medical Research Institute John Hunter Hospital New Lambton Heights Australia; 3 Flinders Health and Medical Research Institute College of Medicine and Public Health Flinders University Bedford Park Australia; 4 School of Health Sciences College of Health, Medicine and Wellbeing The University of Newcastle Callaghan Australia

**Keywords:** stroke, online health information seeking, information-seeking behavior, consumer health information, digital health, eHealth, long-term care, health-risk behaviors, qualitative research, mobile phone

## Abstract

**Background:**

Stroke is a leading cause of death and disability worldwide. As health resources become digitized, it is important to understand how people who have experienced stroke engage with online health information. This understanding will aid in guiding the development and dissemination of online resources to support people after stroke.

**Objective:**

This study aims to explore the online health information–seeking behaviors of people who have experienced stroke and any related barriers or navigational needs.

**Methods:**

Purposeful sampling was used to recruit participants via email between March and November 2022. The sampling was done from an existing cohort of Australian stroke survivors who had previously participated in a randomized controlled trial of an online secondary prevention program. The cohort consisted of people with low levels of disability. Semistructured one-on-one interviews were conducted via phone or video calls. These calls were audio recorded and transcribed verbatim. The data were analyzed by 2 independent coders using a combined inductive–deductive approach. In the deductive analysis, responses were mapped to an online health information–seeking behavior framework. Inductive thematic analysis was used to analyze the remaining raw data that did not fit within the deductive theoretical framework.

**Results:**

A sample of 15 relatively independent, high-functioning people who had experienced stroke from 4 Australian states, aged between 29 and 80 years, completed the interview. A broad range of online health information–seeking behaviors were identified, with most relating to participants wanting to be more informed about medical conditions and symptoms of their own or of a family member or a friend. Barriers included limited eHealth literacy and too much generalization of online information. Online resources were described to be more appealing and more accessible if they were high-quality, trustworthy, easy to use, and suggested by health care providers or trusted family members and friends. Across the interviews, there was an underlying theme of disconnection that appeared to impact not only the participants’ online health information seeking, but their overall experience after stroke. These responses were grouped into 3 interrelated subthemes: disconnection from conventional stroke narratives and resources, disconnection from the continuing significance of stroke, and disconnection from long-term supports.

**Conclusions:**

People who have experienced stroke actively engage with the internet to search for health information with varying levels of confidence. The underlying theme of disconnection identified in the interviews highlights the need for a more comprehensive and sustained framework for support after stroke beyond the initial recovery phase. Future research should explore the development of tailored and relatable internet-based resources, improved communication and education about the diversity of stroke experiences and ongoing risks, and increased opportunities for long-term support.

## Introduction

### Background

Globally, >101 million people are living with the effects of stroke [1]. This population frequently experiences high numbers of unmet needs [2], with unmet information needs being among the most reported [2,3]. The needs of people who have experienced stroke change over time and individuals have different format preferences for information delivery [4]. Both health care providers and people who have experienced stroke have identified that tailored information and support are preferred after stroke [5,6]. With health services and health care providers frequently stretched for time and resources, there is a need to consider alternative or adjunct information and support delivery options, such as internet-based resources, to meet the information needs of this population [7].

Online health information has emerged as an increasingly popular resource, offering people a wealth of knowledge and connection in relation to their health. General population surveys have found up to 86% of people have accessed the internet for the purpose of seeking health information [8]. For 39% of the population in the United States, the internet is the first port of call when seeking health information [9]. Depending on the context, online health information seeking can both positively and negatively influence health-related decision-making [10], medication adherence [11], and relationships with health care providers [12]. In this context, eHealth literacy is an important aspect that determines how people interact with online health information. The term eHealth literacy has been defined by Bautista [13] as involving the interplay of individual and social factors in the use of digital technologies to search, acquire, comprehend, appraise, communicate, and apply health information in all contexts of health care with the goal of maintaining or improving the quality of life throughout the life span. Understanding the factors contributing to health consumers’ eHealth literacy and resulting behaviors will aid in the development of more appropriate online health platforms and content and has also been a growing area of research.

While recent research on online health information–seeking behaviors exists for various health populations, with at least 20 papers published between 2016 and 2021 alone [8], there is a notable gap in the understanding of these behaviors, specifically among people who have had a stroke. Limited existing research provides some surface-level insights into internet access and online health information seeking in people with stroke but leaves many questions unanswered. A cross-sectional study from the United States found that 86% of stroke survivors and their caregivers had access to the internet [14]. An Australian randomized controlled trial of an online stroke secondary prevention program found that 46% of the stroke survivor participants used the internet to access health or medical information at least monthly [15]. In general, the stroke survivor population holds mixed attitudes toward digital health technologies [16]. A segment of this population has no desire to include the internet or technology as part of their own self-management or care [16,17]. Complications after stroke, such as aphasia, can also act as barriers to digital technology use [18]. However, there are people who have experienced stroke who are open to and able to use the internet and technology as one of the tools in their health care management [6,16,17]. While these studies have started to map the attitudes of stroke survivors toward digital health technologies, such studies are often conducted in relation to a specific online program and do not capture the day-to-day online health seeking behaviors of people with stroke.

### This Study

As society continues to digitize health resources, there is a pressing need to understand the day-to-day online health information–seeking behaviors of people who have experienced stroke. This needs to be done to help ensure accurate and relevant information is being appropriately disseminated to meet the information needs of this population. This study aims to address this need by offering the first in-depth exploration of the day-to-day online health information–seeking behaviors, as well as any other navigational needs, of people who have experienced stroke. This will help to guide the future development and dissemination of accurate, relevant, and accessible online health resources related to stroke and reduce the dissemination of unsuitable resources.

## Methods

### Study Design

A qualitative study using a combined inductive–deductive framework analysis approach was used to address the study objectives. A combined inductive-deductive approach has been described by Fereday and Muir-Cochrane [19] and successfully applied in health research [20] in combination with framework analysis [21,22]. This approach was chosen because it allowed for the research questions to be answered against an existing theoretical framework, while leaving space to discover other unexpected aspects of the participants’ experiences [22].

The COREQ (Consolidated Criteria for Reporting Qualitative Research) [23] standards were applied as a guide for research design and for reporting purposes (Multimedia Appendix 1).

### Participants

Participants were recruited from an existing cohort of Australian stroke survivors (n=342) who had previously participated in a randomized controlled trial of an online stroke secondary prevention program, Prevent 2nd Stroke [24,25], between March 2018 and November 2019 and who had consented to be contacted for further studies.

Individuals were eligible to take part in the Prevent 2nd Stroke trial if they were aged ≥18 years, were part of the Australian Stroke Clinical Registry or Hunter Stroke Research Volunteer Registry, experienced stroke 6 to 36 months ago, were sufficiently fluent in English, and had access to the internet via a home device (eg, computer, tablet, or smartphone) or were willing to use public internet services (eg, public library). Individuals were excluded from the trial if they had a Modified Rankin Scale score of ≥4 which indicated that they were unable to walk or attend to bodily needs without assistance. All individuals who consented to being contacted for further studies during the Prevent 2nd Stroke trial were considered eligible for this study, provided they were comfortable completing the interview via telephone call or online video or voice call.

### Procedure

Participants were purposively sampled to recruit a diverse sample based on gender, age, rural or urban location, and reported frequency of access to online health and medical information (frequently, infrequently, or never). The demographic data reported in this study were collected during the Prevent 2nd Stroke trial.

Participants were recruited via email between March and November 2022. The emails contained the participant information statement (Multimedia Appendix 2), consent forms, and the option of receiving a hard copy of these documents via reply paid post. In the consent form, participants experiencing aphasia, or any other condition which limited their ability to participate in the interview, were given the option to nominate a proxy to complete the interview with them. Recruitment ceased once a representative sample of the participants had completed interviews and no new information had emerged from the last 3 participants. Representativeness of the sample was based on age, sex, rural or urban location, and reported access to online health and medical information.

**Figure 1:**
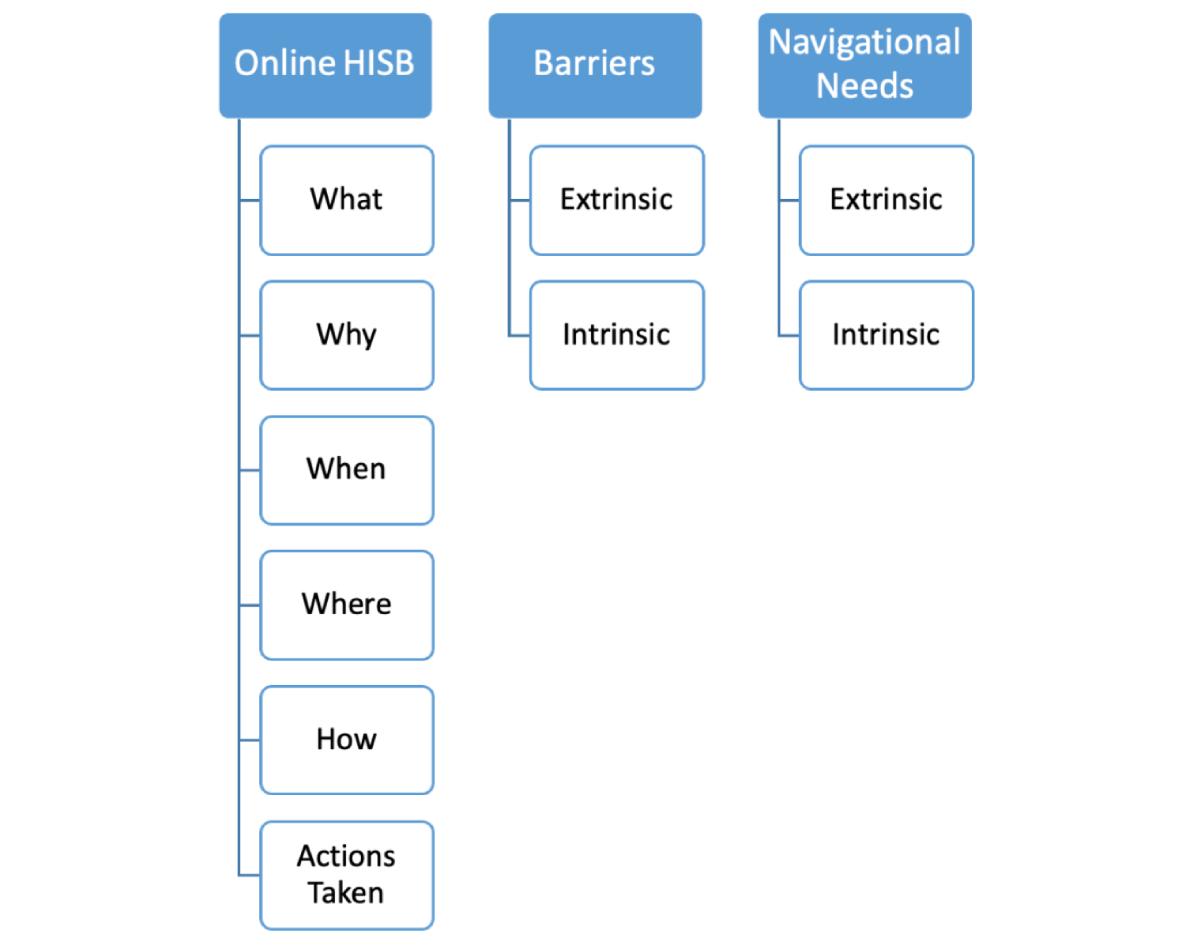
Diagram of the theoretical framework for online health information–seeking behavior (HISB) developed in the study by Lee et al [20].

Two discussion guides were developed for the interviews (Multimedia Appendix 3). At the start of each interview, the following question was posed: “Do you look for health information when you go on the internet?” The participants who answered yes were interviewed using Discussion guide 1 as shown in Multimedia Appendix 3, which was designed to explore the nuance of their online health information–seeking behavior as well as any barriers or navigational needs. Participants who answered no were further prompted to understand whether they had ever used the internet to find health information, before being asked about why they chose not to use the internet for this purpose, their preferred methods for finding health information, and whether they desired for online options to be accessible to them in the future. These discussion guides were developed on the basis of the theoretical framework devised by Lee et al [20] (Figure 1) for exploring the health information–seeking behaviors of people with chronic health conditions.

All interviews were conducted via phone or video call and audio recorded. The deidentified recordings were transcribed verbatim by a third-party transcription service bound by a confidentiality agreement. The translation accuracy was checked by the interviewer and minor grammatical corrections made where they were needed to improve the readability of the transcripts. Participants were given the option to review their interview transcript, and 2 participants opted to receive the transcript but neither made any changes. Interviews ranged between 25 and 75 minutes in length and field notes were taken throughout by the interviewer. One recording from the final third of data collection failed during the interview and field notes were used for analysis instead. No new codes were identified in this interview.

### Reflexivity

A single member of the research team (BC) conducted all interviews. BC is female and was a health and medicine PhD candidate at the time of the interviews. She held a bachelor of health degree, had previous experience leading a qualitative project, and had participated in NVivo training and small-group qualitative research training with an expert who was independent of the study. This research project was part of BC’s PhD thesis on support after stroke and she felt it important to expand on the work she contributed to in the Prevent 2nd Stroke trial by including more nuanced, qualitative perspectives of people with stroke.

BC was the primary contact for the participants during the Prevent 2nd Stroke trial. Contact during the trial was via a study-specific email and phone line and no personal relationships were formed. During the interviews participants were not aware of BC’s personal background or motivations beyond the intentions of the study communicated in the information statement. The ethics approved discussion guide was adhered to when conducting interviews to reduce potential interviewer bias.

### Analysis

A combined inductive–deductive qualitative methodology was used for data analysis [19]. The framework method [22] was applied to the deductive analysis whereby the theoretical framework in the study by Lee et al [20] (Figure 1) was used as the basis for the a priori development of a deductive codebook (Table 1). The examples in the codebook were taken from the findings of the study by Lee et al [20]. Any remaining raw data that did not fit within the deductive codebook were inductively analyzed based on Braun and Clarke’s [26] approach to thematic analysis. The inductive thematic analysis was undertaken with a semantic, realist approach, where codes were developed directly from the data and grouped into themes based on the surface meaning of participant responses.

**Table 1:** Codebook developed a priori, based on the theoretical framework of Lee et al [20], for online health information–seeking behaviors and utilization in the deductive analysis.

Category and code			Scope		Examples
**Online health information**–**seeking behaviors**					
	What	What types of health information are being sought on the web?		Information about medications; lifestyle information; information about specific health professionals, hospitals or practices; disease specific associations, etc.	
	Why	Why are they searching for health information on the web? What is the driving factor?		To be more informed, to clarify things discussed in a consultation, to seek alternative treatment options, self-management, etc.	
	When	When is health information being sought on the web?		Before or after a consultation with a health care professional, when required, etc.	
	Where	Where do consumers go to obtain online health information? What are their sources of information?		Search engines such as Google or Bing, Wikipedia, stroke association websites, forums, journal articles, etc.	
	How	How do consumers go about obtaining health information on the web? What are their search strategies?		Direct link to the website, first page of results from search engine, following recommendations from others, etc.	
	Actions taken	What do consumers actually do with the information they find on the web?		Discuss with a health professional or other people, use it to help decide if they should consult a health professional, trial lifestyle modifications, etc.	
**Barriers to online health information seeking**					
	Extrinsic	External barriers related to the environment or health system		Use of medical jargon, inconsistency of information across sources, volume of information available, availability of content, etc.	
	Intrinsic	Intrinsic barriers pertaining to the individual		Motivation to seek health information, knowledge of medical conditions and their management, limited eHealth literacy, limited knowledge of credible websites, unsure of the information needed, limited time available to search for information, etc.	
**Navigational needs for online health information seeking**					
	Extrinsic	External factors identified as a need or want related to the access of online health information		Greater availability or accessibility of content, single point of contact for information, health professionals providing recommendations, and help from someone to access online information	
	Intrinsic	What an individual possesses within themselves that improves their access, understanding, or use of online health information.		Lee et al [20] did not find any intrinsic factors.	

The combined deductive–inductive approach process was tested by 2 coders (BC and AG) after the completion of 3 interviews to test the fit of the theoretical framework and combined approach for these data. There was a high level of agreement between the coders for the inductive and deductive codes and they opted to continue with the approach.

This approach to analysis was then applied to the remainder of the interview transcripts. This was an iterative process with multiple meetings between the 2 coders to discuss the interpretation of the data and possible groupings and themes. The proposed themes were discussed with the authorship group for triangulation and finalized together to ensure clarity in theme definitions.

All qualitative data were analyzed and organized using NVivo12 (Lumivero) software.

### Ethical Considerations

Ethics approval was granted by the University of Newcastle Human Research Ethics Committee for both the original Prevent 2nd Stroke trial (reference number H-2017-0051) and this qualitative study (reference number H-2021-0410).

Participation was voluntary for all; the participants could withdraw without giving a reason at any given time until data were permanently deidentified. A written informed consent for participation was obtained from all the participants; they were given the option to ask any further questions about the study before and after the consent was obtained. The participants received a gift card worth Aus $20 (US $13.90) as compensation for the time spent. All study data have been deidentified to maintain participant privacy and confidentiality.

## Results

### Overview

A total of 145 participants from the Prevent 2nd Stroke trial were invited to participate in this study. In total, 20 people expressed interest in participating, of whom 15 (75%) consented, enrolled, and completed a 1-on-1 interview. There were 9 (60%) female and 6 (40%) male participants. The age of the participants ranged between 28 and 79 years and they resided in 4 Australian states, with the majority living in metropolitan cities. The demographics of these participants is consistent with the Prevent 2nd Stroke cohort published elsewhere [24], although there appeared to be a higher proportion of females in this sample. Further demographic details can be seen in Table 2.

**Table 2:** Demographic characteristics of interview participants (n=15).

Variable		Participants, n (%)
**Sex**		
	Male	6 (40)
	Female	9 (60)
**Age (y)**		
	≤45	2 (13)
	46-64	5 (33)
	>65	8 (53)
**Rurality**		
	Metropolitan	12 (80)
	Rural	3 (20)
**State**		
	Queensland	8 (53)
	Victoria	4 (27)
	New South Wales	2 (13)
	Western Australia	1 (7)

### Deductive Analysis

Textboxes 1 and 2 outline the responses relating to the online health information–seeking behaviors of the participants. This includes what types of online information were being sought; why, when, where, and how online information was being sought; and the actions taken as a result of the online health information found.

**Participant’s self-reported online health information–seeking behaviors: what, why, and when (responses are listed in the approximate order of frequency from the most to the least frequently mentioned response).**

**What**

- Medical conditions (including looking up symptoms)
- Lifestyle information (diet and exercise information)
- Procedures and associated risks
- Medicines
- Natural health products
- Information about individual health professionals or health services

**Why**

- To be more informed about a medical condition
- To feel worried about something or seeking reassurance
- To seek clarification of information from the participants’ health care providers
- To clarify about something about which the participants have limited knowledge
- To self-manage a medical condition
- Because it is part of the participant’s job (eg, for work-related research or working in health-related field)
- To satisfy the participants’ curiosity for knowledge
- To help or provide family and friends with information

**When**

- After a consultation with a health care professional
- When required
- After being prompted by media (eg, reading an article in a magazine, reading a newsletter, or viewing an advertisement)

**Participant’s self-reported online health information–seeking behaviors: where, how, and actions taken (responses are listed in approximate order of frequency from most to least frequently mentioned).**

**Where**

- Search engines (eg, Google)
- Journal articles
- Peak body or government-based websites
- YouTube
- Social media (eg, Facebook)
- Alternative health websites
- Blogs

**How**

- Start with a fresh search using a search engine.
- Start with websites recommended by others.
- Revisit direct website link.

**Actions taken**

- Be more mindful of health or trial lifestyle modification.
- Discuss with health care providers.
- Discuss with family or friends.
- Decide whether to consult a health care professional or self-manage symptoms.
- Record, download, or otherwise save the information.
- Purchase a health care product.

Participants reported searching for symptoms and medical conditions (*what*). When asked specifically about their online information searching in relation to the 5 risk factors of stroke (ie, diet, physical activity, alcohol use, smoking, and mental health), the participants reported primarily searching for lifestyle information in relation to diet and physical activity. These searches typically involved looking up recipes or exercise routines. However, participants did not indicate that they made any connection between these searches and their perception or understanding of stroke risk factors.

The most common reasons for the participants seeking health information on the internet were so that they could be more informed about a medical condition and to seek reassurance if they felt worried about something. They searched for questions such as the following: “Is this normal?” (*why*). The most common time during which the participants sought online health information was after consulting a health professional (*when*).

Most participants described beginning with a fresh search on a search engine (eg, Google Search) to find their online health information (*where and how*). A third (5/15, 33%) of the sample reported accessing peer-reviewed research journal articles as a source of online health information (*where*). However, in interpreting these data, consideration should be given to the fact that most participants who reported accessing journal articles had a career background in health or research. Information found on the internet prompted the participants to try new wellness activities (eg, trying new exercises and recipes and doing meditations) as well as discussing the information with health care providers, friends, and family. The participants also used the information they found on the Internet to help determine whether to book a consultation with a health care provider or to self-manage symptoms at home (*actions taken*). In the case of 2 (13%) participants, reading the information they found during their online health information searches sometimes made them feel more anxious.

Table 3 reports a summary of the participants’ self-reported barriers to online health information seeking. Common extrinsic barriers included the high volume of information available and how generalized this information tended to be, with participants struggling to find content that they felt was related to their specific health situation. Limitations in eHealth literacy encompassed many of the participants’ individually-reported intrinsic barriers, including difficulties with searching, acquiring, comprehending and appraising online health information. A total of 4 participants did not report any barriers to online health information seeking.

**Table 3:** Participants’ self-reported barriers to seeking online health information.

Category	Feedback
Extrinsic	Online information too generalized (doesn’t feel relevant) Volume of information available Paywalls (eg, to access journals) Poor internet connection
Intrinsic	Limited eHealth literacy Distrust (eg, disliking “Dr Google,” awareness of the high volume of unreliable online sources, fear of scams) Condition-related difficulties (eg, fatigue, memory) Limited time to improve eHealth literacy skills Don’t feel a need for online resources

Table 4 summarizes what participants reported as their navigational needs for finding health information on the internet. The participants expressed “trust in websites” as an important aspect; this trust could be supported by extrinsic factors such as health care providers recommending specific online resources to their patients and the perceived high quality and trustworthiness of websites and online content. Other extrinsic navigational needs that helped to facilitate online health information seeking included access to content that is easy to search and navigate, quick to load and its availability in diverse formats (eg, text and video) and where necessary, receiving support from another person in finding and understanding online health information. Two intrinsic navigational needs arose from the responses. A background in health or research generally appeared to be associated with greater confidence and motivation to access health information on the internet. Participants also reported being more likely to continue returning to online content when it felt personalized and relatable to their individual situation.

**Table 4:** Participants’ self-reported barriers to seeking online health information.

Category	Feedback
Extrinsic	Health care providers suggesting online resources Websites and content that feels “quality” and “rustworthy” Easy to find (eg, first page of results) Receiving help from someone else Search engine suggests related content Helpful links on front page of website Quick loading of web pages Content available in diverse formats
Intrinsic	Background in health or research

### Inductive Analysis

**Figure 2:**
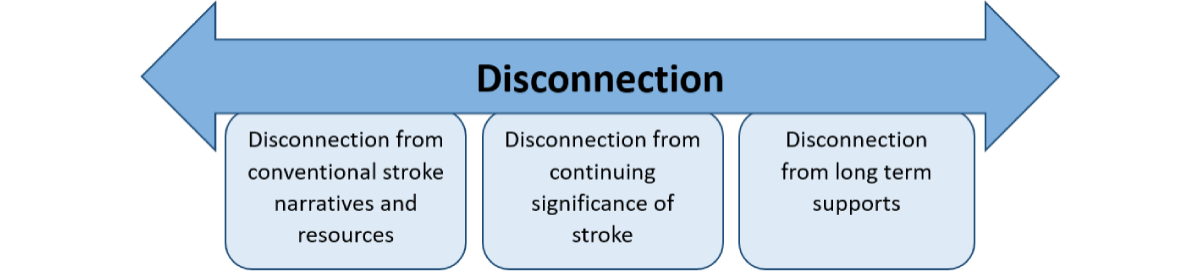
Visual representation of the theme and subthemes identified during inductive thematic analysis of the participant interviews.

The inductive analysis revealed a significant underlying theme of disconnection. This theme presented in varying ways and to different extents throughout the interviews and appeared to impact not only the participants’ online health information–seeking behaviors but their overall experience after stroke. This study grouped these responses into 3 subthemes that had strong interrelations with one another: (1) disconnection from conventional stroke narratives and resources, (2) disconnection from the continuing significance of stroke, and (3) disconnection from long-term supports (Figure 2).

#### Disconnection From Conventional Stroke Narratives and Resources

During the interviews, many of the participants expressed that they did not feel as though they had the “typical” stroke experience. This feeling was accompanied by a lack of information and resources that participants felt were relevant to their individual circumstances. Some participants felt as though they were not represented in the stroke information because they were younger than the usual stroke demographic, or their stroke type was too rare, leading to a sense of exclusion. Others, who stated lifestyle factors were not the cause for their stroke, felt that the available information was tailored to less-healthy individuals and not relevant to them:

I found a lot of the stroke resources were really like not that relevant to me...none of the information really helped, because all the information was lose weight, don’t smoke, don’t drink, but I already didn’t do any of that stuff, so then there’s not anything useful for me to do.

Participant #4

Stroke Australia was moderately helpful for me...I read the online forums...I may even have contributed...But again, I didn’t fit in there. Because I’d had the wrong type of stroke and I was the wrong age and all of that kind of thing. The information was not really helpful to me.

Participant #7

For some participants, it was their comparatively robust recovery that made it challenging to relate to the narratives of other stroke survivors. There was the perception that a full recovery after a stroke was an uncommon event and that it meant they did not “fit in” with other people who had experienced stroke. For some, this extended to a hesitancy in joining stroke groups or in exploring opportunities to connect with other stroke survivors:

I sort of went from nearly dying to walking out, and not many other people have the very similar experience that I had. So, I found stroke groups or anything like that, I didn’t really quite fit in, in a way.

Participant #5

Some participants very actively chose not to dwell on the event and did not want to spend time thinking about the stroke. This affected their willingness to join stroke groups and to be around other people who had experienced stroke and might have experienced more severe effects:

Interviewer: what stopped you from joining any stroke-related groups?

*P* 10: I just didn’t really want to dwell on it. And I just, I kind of knew that in the scheme of things, I wasn’t terribly badly off, and like I thought it would probably be really a bit more depressing, and I didn’t really think it was going to help me in any way.

I’ve never gone to one of those stroke help groups or anything like that, ‘cause, I kind of figure I’m sort of a bit in denial...If you hang around people that are all very seriously affected by stroke, you’ll find little things that will get to you and you will make yourself more sick eventually.

Participant #2

While some people did not want to connect with stroke communities, others expressed a clear interest in connecting with other people who had undergone comparable stroke journeys. They also expressed interest in reading narratives of stroke experiences that more closely resembled their own circumstances:

Stroke survivors’ stories. I think they’re very powerful. Of how to navigate the system, moving forward...and not only just the actual stroke story, but other issues that people come up with afterwards...if that then led to different websites with links...[to] actual factual information, then that would be of benefit I think.

Participant #5

I think that maybe I would have been more likely to do social media and those sorts of things if I knew that the group that I was joining was likely to be someone closer to my age group, rather than potentially say someone 60 or 70.

Participant #10

#### Disconnection From the Continuing Significance of Stroke

Across the interviews, there appeared to be a general disconnection between the stroke event and the perceived relevance of the stroke to the interviewee’s life now. Many participants felt that they were fully recovered now, and that as a result the stroke was no longer relevant to them. While some participants indicated that they searched for online information related to their stroke during the acute stage, only 1 participant reported that they still searched information related to stroke; however, this was infrequent:

The stroke’s something I can’t change.

Participant #2

About my stroke, I haven’t looked up stuff for years. I haven’t needed to because my recovery was so good physically and mentally that it was like “oh well,” [laughs], you know.

Participant #7

Not stroke-related [information searching] I haven’t, because I’ve just, you know, it’s been six years and I’ve just got on with life. You know, I’ve fully recovered from that.

Participant #8

Only 2 participants made any mention of the risk of a recurrent stroke. These mentions were in relation to frustrations about follow-up care and not to their own potential risk of recurrent stroke:

Once you’re out the door initially...there’s nothing to tell you to go back to be monitored. ‘Cause they could nip a lot of things in the bud...so frustrating...And if you don’t have support...there goes your second stroke.

Participant #2

Participants were asked specifically about their online health information seeking related to 5 health-risk behaviors for stroke: diet, physical activity, smoking, alcohol use, and mental health. The participants frequently answered that they searched for topics such as recipes, exercises, or meditations. However, in their responses, the participants did not link these searches as being, in any way, related to their stroke experience or the risk of a recurrent stroke. It was uncommon for the participants to look up anything related to smoking or alcohol.

#### Disconnection From Long-Term Supports

For some participants, there was a strong dissatisfaction with their follow-up experience after stroke. In some cases, follow-up was lacking altogether and there was the perception that you are left on your own after discharge from the hospital. Others experienced frustration around the disorganization in their follow-up and mixed messaging from their health care providers:

With the stroke, once you actually leave hospital, that’s it. [Laughs] That’s all... you’re on your own. I’ve had absolutely no follow-up...in relation to my stroke, none whatsoever...They, they just toss you out the door, and that’s it; you’re on your own.

Participant #8

They sort of stuffed the care up afterwards because they were like, “you do have to have a halter,” “you don’t have to have a halter,” “you don’t need to check this,” “you do,” and “there was no consensus.”

Participant #5

This lack of follow-up and longer-term support caused a significant amount of anxiety for 1 participant. Their account encapsulates the distress that can accompany inadequate follow-up after stroke:

No-one phoned us after being discharged from hospital...then I think after that I got so anxious...I thought, “oh my goodness, am I having another stroke?” And then I would ring up the ambulance and, you know, waste their time a bit...I feel like there’s not a lot of support for that. I just felt anxious, and I couldn’t, I couldn’t plan my life in case something bad was going to happen to me.

Participant #3

There appeared to be a perception that, as opposed to being something that is provided by health care professionals, a lot of the burden was on the stroke survivor to proactively ask questions and book appointments:

Because I think once you’re out the door initially, unless you do make a point of going to the GP...if you don’t have medications there’s, there’s nothing to tell you to go back to be monitored.

Participant #2

The last thing that you want to do is to get yourself into a situation where you actually have another [stroke]...and whenever I go to the doctor’s, that question [about stroke recurrence] is never, ever asked, ever. So, from that point of view it’s, that’s disappointing. It’s up to you to bring it up to them...because the poor buggers are so busy.

Participant #8

When speaking about follow-up, there was a strong desire for some kind of check-in after the participants’ hospital or rehabilitation discharge. This was most often driven by a need for reassurance that their recovery was on the right track:

You think you’re doing the right thing and it would be nice to get confirmation from them [medical professionals] “you may need to change this,” or “you may need to change that,” sort of thing.

Participant #8

## Discussion

### Principal Findings

This study offers new knowledge about the online health information–seeking behaviors in people who have experienced stroke. A broad range of behaviors were identified, with most relating to participants seeking information about medical conditions and symptoms for themselves or someone they know. This is in line with what has previously been studied in people of a similar age range with chronic conditions [20]. This study’s participants faced barriers related to limited eHealth literacy and high volumes of online information. Similar barriers were identified in a recent systematic review on online health information seeking [8]. The condition-related difficulties the participants in this study reported, such as fatigue and memory problems, align with barriers present in general older adult populations [27]. The navigational needs identified centered on the ease of use and support in identifying and accessing high-quality, trustworthy resources. Underlying these behaviors, barriers, and navigational needs, the researchers identified a strong theme of disconnection that was further categorized into (1) disconnection from conventional stroke narratives and resources, (2) disconnection from the continuing significance of stroke, and (3) disconnection from long-term supports.

The subtheme of disconnection from conventional stroke narratives and resources offers some insight into the state of poststroke resources and information provision. The study sample consisted of relatively independent, high-functioning, and “healthy” people who have experienced stroke [24]. Some of our participants reported that internet-based resources were not representative of their experience because they felt too young, too healthy, or too well recovered compared with the typical stroke survivor at whom the information materials were aimed. These feelings also resulted in the participants’ reluctance to access stroke groups and communities on the understanding that they would not fit in or it would not be relevant to their experience. Prior research has found that it is common for people to struggle with identity after a stroke [28-30]. However, this research commonly focuses on how self-perceived identity can change because of stroke, particularly in individuals who experience significant stroke-related disability or disruption to their lives [28,30]. The struggles well-recovered stroke survivors may face in identifying themselves as stroke survivors or relating to stroke resources have not been adequately explored. If there are individuals, such as in this study, who do not feel as though they fit the identity of someone who has had a stroke, this indicates that there may be a significant gap in poststroke information provision and resources. Finding a balance in the narrative of stroke being persistent and disabling [31] as a reflection of the chronic disability up to 50% of people with stroke experience [32] as well as considering stroke as something that some people fully recover from, and that the latter group can still benefit from stroke education, may be beneficial. Everyone experiences stroke differently and has different needs [6], and future development of stroke resources may benefit from greater representation of the diversity of people who experience stroke, and the wide range of outcomes that can be experienced, including full recovery.

Further to their disconnection from the typical stroke narrative, participants in this study generally perceived their stroke as a past event not significant to their current situation. For all participants in this study, the incident of stroke was between 3 and 6 years before the interview. This would likely have a significant bearing on how they perceived the relevance of their stroke. Another qualitative study that included individuals who were between 6 months and 31 years past their stroke found that self-perceived centrality of stroke was related to how significantly the consequences of stroke had impacted their lives [33]. Regardless of the resulting disability, people who have experienced a stroke or transient ischemic attack remain at an increased risk of a recurrent stroke for at least 10 years past the incident event [34]. None of the participants in this study reported looking up stroke–related health-risk behaviors in relation to recurrent stroke risk. Previous studies have shown that there is low awareness of stroke-related health-risk factors among people who have experienced stroke; a significant number of stroke survivors do not receive adequate secondary prevention information [35,36]. If a person who experiences stroke is not receiving appropriate information about the increased risk of subsequent stroke and the health-risk factors that can accompany this risk, there is the concern that feeling disconnected from the stroke event may only further reduce the likelihood of seeking and receiving secondary prevention support. The theme of disconnection from the continuing significance of stroke highlights the importance of providing education about the ongoing risks related to stroke recurrence for people who have experienced stroke.

While the focus of this study was on online health information–seeking behaviors, the issue of inadequate support after stroke was raised by the participants. The participants reported a desire for more check-in and follow-up opportunities with health care providers to provide reassurance that they were on the right track in their stroke-recovery journey, even if they experienced little-to-no ongoing disability. Their responses are in line with previous literature that reported inadequate support received by stroke survivors [5,37] and their significant number of unmet needs [2,3]. As reported by 1 of our participants, the lack of follow-up can be a source of anxiety and can substantially affect their quality of life and increase the pressure on health care systems. The experiences shared here underscore the critical need for enhanced ongoing support mechanisms that address postacute concerns and anxieties of stroke survivors and facilitate a more informed and secure transition to life after stroke.

The number of stroke survivors who are comfortable using technology as part of their recovery and who seek health information is likely to increase [15]. To account for this, future research should investigate various means of creating tailored internet-based resources that can meet the diversity of poststroke needs [6,37]. These internet-based resources would benefit from being cocreated with the stroke community to ensure that they truly meet the needs of their end users [38]. Health care providers are well positioned to be the means of dissemination of these resources by recommending them to patients [39]. Ideally, internet-based stroke resources can be part of a larger long-term care framework that includes multimodal resources and follow-up check-ins from health care professionals.

### Strengths and Limitations

This study used a combined inductive–deductive framework analysis approach [22]. This robust qualitative approach enabled the researchers to effectively answer the research questions using the predetermined framework, allowing for unexpected themes to arise to further enrich the findings. Participants were recruited from the Prevent 2nd Stroke sample [24] which required a modified Rankin Scale score of ≤3 and sufficient access to the internet and an email address. This limits the generalizability of the findings to the wider stroke population as it excludes people who have experienced more significant enduring disability and those who would not meet the internet and email accessibility criteria. The recruitment for this qualitative study also occurred via email invitation, which would likely have further increased the recruitment of people who are more engaged with the internet. The purposive sampling approach, however, did allow for the capture of a diverse range of perspectives within the subgroup of relatively well people who have experienced stroke. The study did not record occupation or educational background in the data captured; it is possible that the people who agreed to participate in this study were more likely to be of a health or research background than the rest of the sample that was considered for recruitment. This possibly had an impact on the number of participants who reported using journal articles as an information source and those who had a background in health or research as a navigational need.

### Conclusions

People who have experienced stroke actively engage with the internet to search for online health information with varying levels of confidence. In analyzing the responses on online health information–seeking behaviors, barriers, and navigational needs, the researchers identified a clear underlying theme of disconnection. The subthemes of disconnection represent some of the challenges people can face after stroke and the need for a more comprehensive and sustained framework of long-term support which recognizes the multifaceted needs of stroke survivors and fosters a cohesive approach to health care management beyond the initial recovery phase. By integrating more tailored and relatable online resources, improved communication and education about stroke and ongoing risks, and increased opportunities for long-term support, our health care systems can foster a more connected, informed, and empathic environment for people who have experienced stroke.

## Data Availability

The datasets generated and analyzed during this study are not publicly available to protect participant privacy, but nonidentifiable data are available from the corresponding author upon reasonable request.

## Appendix

Multimedia Appendix 1 Completed COREQ (Consolidated Criteria For Reporting Qualitative Research) checklist.

Multimedia Appendix 2 Participant information statement.

Multimedia Appendix 3 Discussion guides.

## References

[1] GBD 2019 Stroke Collaborators (2021). Global, regional, and national burden of stroke and its risk factors, 1990-2019: a systematic analysis for the Global Burden of Disease Study 2019. Lancet Neurol.

[2] Lin BL, Mei YX, Wang WN, Wang SS, Li YS, Xu MY, Zhang ZX, Tong Y (2021). Unmet care needs of community-dwelling stroke survivors: a systematic review of quantitative studies. BMJ Open.

[3] Chen T, Zhang B, Deng Y, Fan J, Zhang L, Song F (2019). Long-term unmet needs after stroke: systematic review of evidence from survey studies. BMJ Open.

[4] Du HS, Ma JJ, Li M (2016). High-quality health information provision for stroke patients. Chin Med J (Engl).

[5] Bally EL, Cheng D, van Grieken A, van Dam-Nolen DH, Macchione S, Sanz MF, Carroll Á, Roozenbeek B, Dippel DW, Raat H (2023). A qualitative study of the values, needs, and preferences of patients regarding stroke care: the ValueCare study. Int J Integr Care.

[6] Finch E, Minchell E, Cameron A, Jaques K, Lethlean J, Shah D, Moro C (2022). What do stroke survivors want in stroke education and information provision in Australia?. Health Soc Care Community.

[7] Filip R, Gheorghita Puscaselu R, Anchidin-Norocel L, Dimian M, Savage WK (2022). Global challenges to public health care systems during the COVID-19 pandemic: a review of pandemic measures and problems. J Pers Med.

[8] Jia X, Pang Y, Liu LS (2021). Online health information seeking behavior: a systematic review. Healthcare (Basel).

[9] Finney Rutten LJ, Blake KD, Greenberg-Worisek AJ, Allen SV, Moser RP, Hesse BW (2019). Online health information seeking among US adults: measuring progress toward a healthy people 2020 objective. Public Health Rep.

[10] Bussey LG, Sillence E (2019). The role of internet resources in health decision-making: a qualitative study. Digit Health.

[11] Lim HM, Dunn AG, Lim JR, Abdullah A, Ng CJ (2022). Association between online health information-seeking and medication adherence: a systematic review and meta-analysis. Digit Health.

[12] Tan SS, Goonawardene N (2017). Internet health information seeking and the patient-physician relationship: a systematic review. J Med Internet Res.

[13] Bautista JR (2015). From solving a health problem to achieving quality of life: redefining eHealth literacy. J Lit Technol.

[14] Naqvi IA, Montiel TC, Bittar Y, Hunter N, Okpala M, Johnson C, Weiner MG, Savitz S, Sharrief A, Beauchamp JE (2021). Internet access and usage among stroke survivors and their informal caregivers: cross-sectional study. JMIR Form Res.

[15] Clancy B, Bonevski B, English C, Baker AL, Turner A, Magin P, Pollack M, Callister R, Guillaumier A (2022). Access to and use of internet and social media by low-morbidity stroke survivors participating in a national web-based secondary stroke prevention trial: cross-sectional survey. J Med Internet Res.

[16] Bally EL, Cheng D, van Grieken A, Ferri Sanz M, Zanutto O, Carroll A, Darley A, Roozenbeek B, Dippel DW, Raat H (2023). Patients' perspectives regarding digital health technology to support self-management and improve integrated stroke care: qualitative interview study. J Med Internet Res.

[17] Narbutaitienė J, Björklund Carlstedt A, Fischl C (2024). Stroke survivors' experiences and meaning of digital technology in daily life: a phenomenological study. Disabil Rehabil Assist Technol.

[18] Menger F, Morris J, Salis C (2020). The impact of aphasia on internet and technology use. Disabil Rehabil.

[19] Fereday J, Muir-Cochrane E (2016). Demonstrating rigor using thematic analysis: a hybrid approach of inductive and deductive coding and theme development. Int J Qual Methods.

[20] Lee K, Hoti K, Hughes JD, Emmerton L (2014). Dr Google and the consumer: a qualitative study exploring the navigational needs and online health information-seeking behaviors of consumers with chronic health conditions. J Med Internet Res.

[21] Redman M, Pearce J, Gajebasia S, Johnson M, Finn G (2017). Care of the dying: a qualitative exploration of foundation year doctors' experiences. Med Educ.

[22] Gale NK, Heath G, Cameron E, Rashid S, Redwood S (2013). Using the framework method for the analysis of qualitative data in multi-disciplinary health research. BMC Med Res Methodol.

[23] Tong A, Sainsbury P, Craig J (2007). Consolidated criteria for reporting qualitative research (COREQ): a 32-item checklist for interviews and focus groups. Int J Qual Health Care.

[24] Guillaumier A, Spratt NJ, Pollack M, Baker A, Magin P, Turner A, Oldmeadow C, Collins C, Callister R, Levi C, Searles A, Deeming S, Clancy B, Bonevski B (2022). Evaluation of an online intervention for improving stroke survivors' health-related quality of life: a randomised controlled trial. PLoS Med.

[25] Guillaumier A, McCrabb S, Spratt NJ, Pollack M, Baker AL, Magin P, Turner A, Oldmeadow C, Collins C, Callister R, Levi C, Searles A, Deeming S, Wynne O, Denham AM, Clancy B, Bonevski B (2019). An online intervention for improving stroke survivors' health-related quality of life: study protocol for a randomised controlled trial. Trials.

[26] Braun V, Clarke V (2006). Using thematic analysis in psychology. Qual Res Psychol.

[27] Zhao YC, Zhao M, Song S (2022). Online health information seeking behaviors among older adults: systematic scoping review. J Med Internet Res.

[28] Taubner H, Hallén M, Wengelin Å (2019). Still the same?: self-identity dilemmas when living with post-stroke aphasia in a digitalised society. Aphasiology.

[29] Lapadatu I, Morris R (2019). The relationship between stroke survivors' perceived identity and mood, self-esteem and quality of life. Neuropsychol Rehabil.

[30] Martin-Saez MM, James N (2021). The experience of occupational identity disruption post stroke: a systematic review and meta-ethnography. Disabil Rehabil.

[31] Gorelick PB (2019). The global burden of stroke: persistent and disabling. Lancet Neurol.

[32] Donkor ES (2018). Stroke in the 21 century: a snapshot of the burden, epidemiology, and quality of life. Stroke Res Treat.

[33] Hutton L, Ownsworth T (2019). A qualitative investigation of sense of self and continuity in younger adults with stroke. Neuropsychol Rehabil.

[34] Mohan KM, Wolfe CD, Rudd AG, Heuschmann PU, Kolominsky-Rabas PL, Grieve AP (2011). Risk and cumulative risk of stroke recurrence. Stroke.

[35] Ellis C, Barley J, Grubaugh A (2013). Poststroke knowledge and symptom awareness: a global issue for secondary stroke prevention. Cerebrovasc Dis.

[36] (2020). National stroke audit: rehabilitation services report 2020. Stroke Foundation.

[37] Kjörk EK, Gunnel C, Lundgren-Nilsson Å, Sunnerhagen KS (2019). Experiences, needs, and preferences for follow-up after stroke perceived by people with stroke and healthcare professionals: a focus group study. PLoS One.

[38] Ramage ER, Burke M, Galloway M, Graham ID, Janssen H, Marsden DL, Patterson AJ, Pollack M, Said CM, Lynch EA, English C (2022). Fit for purpose. Co-production of complex behavioural interventions. A practical guide and exemplar of co-producing a telehealth-delivered exercise intervention for people with stroke. Health Res Policy Syst.

[39] van de Graaf DL, Vlooswijk C, Bol N, Krahmer EJ, Bijlsma R, Kaal S, Sleeman SH, van der Graaf WT, Husson O, van Eenbergen MC (2023). AYAs' online information and eHealth needs: a comparison with healthcare professionals' perceptions. Cancer Med.

