# Supplementary material for: The Online Health Information–Seeking Behaviors of People Who Have Experienced Stroke: Qualitative Interview Study
Source: JMIR Form Res. 2024 Oct 18;8:e54827. doi: 10.2196/54827 (PMC11530730; doi:10.2196/54827)
Supplement: Multimedia Appendix 1 [file formative_v8i1e54827_app1.pdf]

No	Item	Guide questions/description	Location
**Domain 1: Research team and reflexivity**			
Personal Characteristics			
1.	Interviewer/facilitator	Which author/s conducted the interview or focus group?	Methodology; reflexivity
2.	Credentials	What were the researcher's credentials? *E.g. PhD, MD*	Methodology; reflexivity
3.	Occupation	What was their occupation at the time of the study?	Methodology; reflexivity
4.	Gender	Was the researcher male or female?	Methodology; reflexivity
5.	Experience and training	What experience or training did the researcher have?	Methodology; reflexivity
Relationship with participants			
6.	Relationship established	Was a relationship established prior to study commencement?	Methodology; reflexivity
7.	Participant knowledge of the interviewer	What did the participants know about the researcher? e.*g. personal goals*, *reasons for doing the research*	Methodology; reflexivity
8.	Interviewer characteristics	What characteristics were reported about the interviewer/facilitator? e.g. *Bias*, *assumptions*, *reasons and interests in the research topic*	Methodology; reflexivity

No	Item	Guide questions/description	Location
**Domain 2: study design**			
Theoretical framework			
9.	Methodological orientation and Theory	What methodological orientation was stated to underpin the study? *e.g. grounded theory, discourse analysis, ethnography, phenomenology, content analysis*	Methodology; study design, procedure, analysis
Participant selection			
10.	Sampling	How were participants selected? *e.g. purposive, convenience, consecutive, snowball*	Methodology; participants, procedure
11.	Method of approach	How were participants approached? e.*g. face-to-face, telephone, mail, email*	Methodology; procedure
12.	Sample size	How many participants were in the study?	Results; overview
13.	Non-participation	How many people refused to participate or dropped out? Reasons?	Results; overview
Setting			
14.	Setting of data collection	Where was the data collected? e.*g. home, clinic, workplace*	Methodology; procedure

No	Item	Guide questions/description	Location
15.	Presence of non-participants	Was anyone else present besides the participants and researchers?	Results; overview
16.	Description of sample	What are the important characteristics of the sample? *e.g. demographic data, date*	Results; overview
Data collection			
17.	Interview guide	Were questions, prompts, guides provided by the authors? Was it pilot tested?	Methodology; procedure
18.	Repeat interviews	Were repeat interviews carried out? If yes, how many?	No – not explicitly stated in manuscript but inferred
19.	Audio/visual recording	Did the research use audio or visual recording to collect the data?	Methodology; procedure
20.	Field notes	Were field notes made during and/or after the interview or focus group?	Methodology; procedure
21.	Duration	What was the duration of the interviews or focus group?	Methodology; procedure
22.	Data saturation	Was data saturation discussed?	Methodology; procedure
23.	Transcripts returned	Were transcripts returned to participants for comment and/or correction?	Methodology; procedure
**Domain 3: analysis and findings**z			

No	Item	Guide questions/description	Location
Data analysis			
24.	Number of data coders	How many data coders coded the data?	Methodology; analysis
25.	Description of the coding tree	Did authors provide a description of the coding tree?	Methodology; analysis
26.	Derivation of themes	Were themes identified in advance or derived from the data?	Methodology; analysis
27.	Software	What software, if applicable, was used to manage the data?	Methodology; analysis
28.	Participant checking	Did participants provide feedback on the findings?	No
Reporting			
29.	Quotations presented	Were participant quotations presented to illustrate the themes / findings? Was each quotation identified? e.*g. participant number*	Results
30.	Data and findings consistent	Was there consistency between the data presented and the findings?	Results
31.	Clarity of major themes	Were major themes clearly presented in the findings?	Results
32.	Clarity of minor themes	Is there a description of diverse cases or discussion of minor themes?	Results
