# Supplementary material for: The Online Health Information–Seeking Behaviors of People Who Have Experienced Stroke: Qualitative Interview Study
Source: JMIR Form Res. 2024 Oct 18;8:e54827. doi: 10.2196/54827 (PMC11530730; doi:10.2196/54827)
Supplement: Multimedia Appendix 2 [file formative_v8i1e54827_app2.pdf]

Dr Ashleigh Guillaumier
College of Health, Medicine and Wellbeing
University of Newcastle
1 University Drive
Callaghan NSW 2308
Ashleigh.Guillaumier@newcastle.edu.au

THE UNIVERSITY OF NEWCASTLE AUSTRALIA

Participant Information Statement for the Research Project:

The online health information seeking behaviours of Australians who have experienced stroke: A qualitative study

You are invited to participate in the research project identified above which is being conducted by Brigid Clancy, a PhD candidate from the School of Medicine and Public Health at the University of Newcastle. The research is part of Brigid's studies at the University of Newcastle, supervised by Dr Ashleigh Guillaumier (School of Medicine and Public Health, University of Newcastle), Professor Billie Bonevski (College of Medicine and Public Health, Flinders University), and Professor Coralie English (School of Health Sciences, University of Newcastle).

Why is the research being done?

Previous research has shown that many people who have experienced stroke lack information or have unanswered questions about their health and stroke generally. Some people look to the internet to find these answers, but nearly 40% do not find all of the information they are looking for.

This study will gather information on how people who have experienced stroke access health and medical information online. This includes what information is looked for, how it is found, why the information is looked for, what is being done with the information once it is found, and if there are any barriers or additional needs to find the information online.

Understanding this will help us to make sure that in the future, online health information is easy to access and answers all the questions of people who have experienced stroke.

Who can participate in the research?

We are contacting you because you participated in a research study with us between 2018-2019 called Prevent 2nd Stroke. During the final survey you answered that you would be willing to be invited to future research.

You are eligible to participate if you received this invitation.

Please note you can nominate a carer, friend or family member to help you participate if you experience any communication difficulties.

What would you be asked to do?

If you agree to participate in this study, you will be asked to sign the Participant Consent form and return it directly to the research team either electronically or by mail. From there, our research team will arrange a time for you to participate in an interview over the phone or via online video call through Zoom at your preference. Zoom's privacy statement can be viewed here https://explore.zoom.us/en/privacy/.

During the interview, you will be asked to answer questions about your experiences and perceptions of online health information. The interview is expected to take between 30 minutes and one hour.

The interviews will be recorded by the research team and a third-party professional transcription service will transcribe the de-identified interviews. You may be quoted verbatim in published materials. However, no identifiable details will be included in the quote. You will be given the opportunity to review the recorded interview and/or transcription. At completion of the interview you will receive a $20 gift card as a thank you for your time.

What choice do you have?

Participation in this research is entirely your choice. Only those people who give their informed consent will be included in the project. Whether or not you decide to participate, your decision will not disadvantage you.

If you do decide to participate, you may withdraw from the project at any time without giving a reason and have the option of withdrawing your data up until data has been published.

How much time will it take?

The interview will take approximately 30 minutes to one hour to complete in total.

To reduce the chance of fatigue this can be done in a single sitting or over multiple sessions at your preference. Just let the research team know what you would prefer.

What are the risks and benefits of participating?

As interviews may take up to an hour you may feel fatigued from participating. You have the option of completing the interview over multiple sessions. This can be decided before or at any point during the interview. Reflecting on your stroke experience and interactions with the health system may cause some discomfort. Carefully consider your participation if you feel you may be at higher risk discomfort or fatigue while participating. If discussing these experiences raises issues for you, call BeyondBlue (1300 22 4636) for support and advice.

The results we get from this study will assist us in making sure that online health information is easy to access and addresses the needs of people who have experienced stroke. However, we cannot promise any individual benefit from participating in this research.

How will your privacy be protected?
All personally identifiable information collected from you for the study will be treated confidentially. Access to any identifiable data is restricted to the research team named above, unless disclosure is required by law in order for us to comply with our regulatory obligations. Recordings will be transcribed by a professional transcription service bound by a confidentiality agreement. All electronic information will be stored in password protected files on a secure, University-hosted online platform, with access available only to authorised research team members. All data will be managed/stored in accordance with the University's Research Data and Materials Management Guideline (see https://policies.newcastle.edu.au/document/view-current.php?id=72) or any successor Guideline, and applicable University of Newcastle policy provisions (as amended from time to time). At the end of the study, all information will be stored securely for at 5 years at the University of Newcastle, after which time all electronic information permanently deleted.

How will the information collected be used?

The information collected will be published as a paper in a scientific journal. It will also be submitted as part of Ms Brigid Clancy's PhD. It may also generate other publications and presentations that aim to inform the accessibility of online health information resources for people who have experienced stroke.

You will be able to review the recording and/or transcripts from the interview on request and will be reminded of this option at the end of the interview. You will be able to edit or erase your contribution on review of these files if you choose to.

Non-identifiable data may be also be shared with other parties to encourage scientific scrutiny, and to contribute to further research and public knowledge.

If you would like a copy of the summary of the results, please let the research team know during the interview or contact Brigid Clancy after July 2022 at Brigid.Clancy@newcastle.edu.au.

What do you need to do to participate?

Please read this Information Statement and be sure you understand its contents before you consent to participate. If there is anything you do not understand, or you have questions, contact the researcher.

If you would like to participate, please email your completed consent form to Brigid.Clancy@newcastle.edu.au. You can also request that a hard copy and a reply-paid envelope to be sent to you to be sent back via mail. You will then be contacted to arrange a time convenient to you for the interview.

Further information

If you would like further information please contact:

Ms Brigid Clancy

Brigid.clancy@newcastle.edu.au

Or

Dr Ashleigh Guillaumier

Ashleigh.Guillaumier@newcastle.edu.au

Thank you for considering this invitation.

Ashleigh Guillaumier
Research Fellow

Brigid Clancy
PhD Candidate

Complaints about this research

This project has been approved by the University's Human Research Ethics Committee, Approval No. H-2021-0410.

Should you have concerns about your rights as a participant in this research, or you have a complaint about the manner in which the research is conducted, it may be given to the researcher, or, if an independent person is preferred, to the Human Research Ethics Officer, Research & Innovation Services, The University of Newcastle, University Drive, Callaghan NSW 2308, Australia, telephone (02) 4921 6333, email Human-Ethics@newcastle.edu.au.
