# Supplementary material for: The Online Health Information–Seeking Behaviors of People Who Have Experienced Stroke: Qualitative Interview Study
Source: JMIR Form Res. 2024 Oct 18;8:e54827. doi: 10.2196/54827 (PMC11530730; doi:10.2196/54827)
Supplement: Multimedia Appendix 3 [file formative_v8i1e54827_app3.docx]

# Multimedia Appendix 1

## The online health information seeking behaviours of Australians who have experienced stroke: A qualitative study – Discussion Guide

Document version 2: 16 December 2021

Hi [participant name],

My name is Brigid. I am a researcher at the University of Newcastle.

This interview is being conducted so that I can learn more about if and how you find health information online, and any problems you experience. The interview will take around 30 minutes to an hour depending on your answers. There’s no right or wrong answers, I’m interested in your opinions and experiences. I do want to assure you that your opinions will be very welcome. Although I’ll be asking you some specific questions to guide our chat today, we can also explore any other aspects of your experiences searching for health information that you would like to discuss.

To begin with I’d just like to go over some key things from the information statement. Firstly, this interview will be recorded to ensure that I get all the details but will be able to pay full attention to you during the interview. All of your comments will remain confidential. I will be compiling a report with comments from all of the interviews I’ve conducted, but you will remain anonymous. Most importantly you can stop at any time and if you want to have any comments deleted just let me know.

I will also check in to see how you are going throughout the interview and you are welcome to take a break or continue the interview at another time if it is too fatiguing.

Did you have any questions that you would like to ask me?

**Introductory question**

Do you look for health information when you go on the internet?

Yes – use discussion guide 1

No – use discussion guide 2

**Discussion guide 1**

1. What kinds of health information do you look for when you go on the Internet?
   - PROMPT: Information about health conditions, health services available, information about medicines, for yourself or other people.
   - Example follow up: Do you look for any other kinds of health information online? If so, what are they? Primarily stroke-related or other conditions?
2. There’s a few more specific topics I’d like to see if you have looked up online since having your stroke: diet, physical activity, alcohol, smoking, mental health?
   - PROMPT: why did you look it up? (in relation to stroke risk?)
3. When do you go online to find health information?
   - PROMPT: before/after an appointment, just generally if something doesn’t feel right, looking up information for friends or family members
   - Follow up: do you normally find what you are looking for? Why/why not?
4. Do you ever have difficulties when using the internet to find health information?
   - What sort of difficulties?
   - Example follow up question: do you seek help when you do experience difficulties? Why/why not?
   - If they do seek help, who do they seek help from?
5. What do you do with the information you find online?
   - PROMPT: discuss it with a healthcare provider, try new things, discuss it with family/friends
6. How do you find the health information you are looking for on the internet?
   - PROMPT: do you use search engines? Do you go to a particular website? Do you use or post questions on forums or social media?
   - Follow up: How did you find out about these resources?

***Fatigue check in reminder***

1. Are there any apps or online resources that you have used more than once?
   - PROMPT: any websites you regularly use, any apps for tracking health
   - Example follow up questions: What made you want to keep using it? Are you still using it? What made you stop using it?
2. Has a healthcare provider ever recommended websites or apps to you related to your stroke or any other conditions?
   - Example follow up for yes: What were they? Did you access them? Were they useful?
   - Example follow up for no: Would you have liked them to? What sort of resources would you have liked them to refer you to?
3. How satisfied do you usually feel with the health information or resources you find online?
   - PROMPT: Do you feel that you’ve found what you’re looking for?
   - Follow up question: Can you think of anything that would help you find what you want to find? PROMPT: help from a person or computer program? What would make it easier for you to find health information on the internet?
4. For someone else who has had a stroke, what websites would you direct them to?
   - What is it about those websites that makes you want to direct others to it?
5. Where else do you get your health and/or stroke related information from?
   - PROMPT: healthcare professionals, pamphlets, friends, family, community stroke groups
   - Follow up: What is your preferred way to get this information?
6. What information or resources would you like to see available to stroke survivors in the future?
   - Follow up: how would you like to hear about them? How would you like to access them?

**Discussion guide 2**

Prompt again to see if they have ever looked for health information on the internet. Ask specifically what their experience was, and ask relevant questions from discussion guide 1 about their past experience, and discussion guide two about why they no longer use the internet for this purpose.

1. What has stopped you from looking for health information on the internet?
   - PROMPT: lack of interest, not knowing how to search for information, lack of resources (e.g. no device to access it on)
2. Where do you prefer to get your health information from?
   - PROMPT: healthcare professionals, pamphlets, friends, family, community stroke groups
   - Follow up: Are you satisfied with the information? Does it answer all of your questions?
   - Follow up: What do you do if you have a question that hasn’t been answered by the above answers?
3. Has a healthcare provider ever recommended websites or apps to you related to your stroke or any other conditions?
   - Example follow up for yes: What were they? Did you access them? Were they useful?
   - Example follow up for no: Would you have liked them to? What sort of resources would you have liked them to refer you to?
4. There’s a few particular topics I’d like to see if you have accessed information about from any source (e.g. doctor, family, friends, community groups): diet, physical activity, alcohol, smoking, mental health?
   - PROMPT: why did you look it up? (in relation to stroke risk?)

***Fatigue check in reminder***

1. Is using the internet for searching for health information or accessing health or stroke related resources something you would like to do?
   - If yes: What sort of resources or information would you like to access? How would you like to be supported to do this?
2. For someone else who has had a stroke, what websites would you direct them to?
   - What is it about those websites that makes you want to direct others to it?
3. What information or resources would you like to see available to stroke survivors in the future?
   - Follow up: how would you like to hear about them? How would you like to access them?

**Conclusion**

Thank you for taking the time to speak with me today. I would like to express how appreciative we are of you sharing your thoughts and experiences. Is there anything else you would like to add?

Would you like to review either the audio recording or the transcript of this interview? This will give you the opportunity to edit any comments you have made or withdraw the interview if you are unhappy with it.

Thank you again for all of your comments.
